# Tegumentary Leishmaniasis in Northeastern Italy from 2017 to 2020: A Neglected Public Health Issue

**DOI:** 10.3390/ijerph192316047

**Published:** 2022-11-30

**Authors:** Valeria Gaspari, Tommaso Gritti, Margherita Ortalli, Annalisa Santi, Giorgio Galletti, Arianna Rossi, Gianluca Rugna, Andrea Mattivi, Giulio Matteo, Gian L. Belloli, Giovanna Mattei, Stefania Varani, on behalf of the Skin_Leish_RER Network

**Affiliations:** 1 Unit of Dermatology, Head and Neck Department, IRCCS Azienda Ospedaliero-Universitaria di Bologna, 40138 Bologna, Italy; 2 Section of Microbiology, Department of Experimental, Diagnostic and Specialty Medicine, University of Bologna, 40138 Bologna, Italy; 3 Istituto Zooprofilattico Sperimentale della Lombardia e dell’Emilia Romagna, 25124 Brescia, Italy; 4 Regional Health Authority, Emilia-Romagna Region, 40127 Bologna, Italy; 5 Unit of Microbiology, IRCCS Azienda Ospedaliero-Universitaria di Bologna, 40138 Bologna, Italy

**Keywords:** *Leishmania infantum*, tegumentary leishmaniasis, cutaneous leishmaniasis, mucosal leishmaniasis, autochthonous cases

## Abstract

Tegumentary leishmaniasis (TL) includes cutaneous (CL) and mucosal (ML) leishmaniasis; despite being endemic in southern Europe, it is often underdiagnosed and underreported. This study aimed to retrospectively examine data collected from patients with TL in a selected area of northeastern Italy (Emilia-Romagna region, RER). A network of 10 diagnostic units within RER was established, and TL cases diagnosed in RER from 2017 to 2020 were evaluated. A total of 135 TL cases were collected (62% male, 38% female); patients ranged from 1 to 84 years, with a median age of 57. Among these cases, 113 (84%) were notified to the public health authorities. The average annual incidence of TL was 0.76 cases per 100,000 inhabitants. Infections were acquired within the RER in 84% of cases; the 113 autochthonous cases were distributed in the foothills areas of the region. We provide evidence of a defined spatial distribution of TL cases in a selected area of northeastern Italy, as well as a relevant number of ML cases. Our observations suggest the need to raise awareness about TL among clinicians and pathologists, promote the molecular confirmation of cases by reference laboratories, and encourage the establishment of surveillance networks for this neglected disease.

## 1. Introduction

Leishmaniases are a group of vector-borne diseases caused by protozoan parasites of the genus *Leishmania* and transmitted by phlebotomine sand flies (Diptera: Psychodidae) [1]. The clinical spectrum of the disease depends on parasite species and host immune response, varying from visceral leishmaniasis (VL) to tegumentary leishmaniasis (TL), which includes cutaneous leishmaniasis (CL), mucosal leishmaniasis (ML), and mucocutaneous leishmaniasis (MCL) [2].

CL is the most common form of the disease, with a global incidence of around 1 million cases/year [3]. It has been estimated that around 10% of uncomplicated CL cases progress to mucosal disease, diffuse CL, or disseminated CL, mainly depending on the parasite species and/or the host immune system [4]. Around 207,000 CL cases were reported to WHO in 2020; most cases were recorded in the Eastern Mediterranean region and American region, while only 971 cases were displayed in the European region [5,6].

TL is a neglected disease in Europe; it is often misdiagnosed or diagnosed with delay, and its surveillance is not mandatory in Austria, Belgium, France, and Germany [7]. The CL underreporting rate has been estimated to be 2.8–4.6 fold in the Mediterranean basin [1,8] by considering the number of reported CL cases/year as compared to the estimated annual CL incidence in each country, ML being even more underrecognized by clinicians than CL [1,9].

*Leishmania infantum* is the causative agent of canine leishmaniasis (CanL) in the Mediterranean basin as well as human VL and TL [4,10]. ML lesions caused by *L. infantum* are not clearly reported to be associated with previous CL [2]; the chronic infections develop into nodules or polypoid, infiltrating or ulcerated lesions, predominantly into the buccal area, the pharyngeal and laryngeal area, and the nose [9,11].

In Italy, case notification for human leishmaniasis is mandatory, but a high rate of undernotification is estimated as TL is often a benign disease that does not require hospitalization [1]. In 2018, the first report on the epidemiological situation of Italy on human leishmaniasis was published by the WHO; between 1998 and 2016, only 800 cases of CL were notified all over the country [12]. CL cases classically occur in the Tyrrhenian littoral, southern peninsular regions, and the main islands [13]. 

Nevertheless, the epidemiological pattern of leishmaniasis in Italy is changing, leading to an increase in human cases in the northern part of the country. An upsurge of CL cases was recently observed in the Emilia-Romagna region (RER) in northeastern Italy, with clusters in areas between the provinces of Modena and Bologna and in the provinces of Forlì-Cesena and Rimini [14]. A study performed in the Bologna province showed a raised incidence of CL cases in 2013–2015; the incidence of CL was 0.50/100,000 in 2013, 1.49/100,000 in 2014, and 1.00/100,000 in 2015, respectively, as compared to an average incidence of 0.12/100,000 in 2008–2012, with a four- to twelve-fold increase in comparison to the previous years [15]. In line with these findings, an increased number of CL cases (n = 21) was diagnosed in 2014–2016 at the Pathology Unit of Modena, which accounted for 60% of all cases that were diagnosed in a 20-year period (n = 35) (1997–2016) in the same unit [16].

In 2014, the upsurge of human leishmaniasis cases in RER [14,15,16,17,18] led to the establishment of a regional reference laboratory (RRL) to confirm human cases by molecular methods [19,20]; the RRL complemented a previously developed program for serological surveillance of CanL and entomological surveillance of vector-borne diseases [10]. 

Despite the strengthening of the surveillance system, underdiagnosis and undernotification of TL cases are still a problem in RER. This study aimed to retrospectively examine the cases of TL identified in RER from 2017 to 2020. To ameliorate the evaluation of TL cases within RER, a network of 10 diagnostic units was established. Each unit was composed of a multidisciplinary team of dermatologists, infectious disease specialists, pathologists, parasitologists, and epidemiologists. 

## 2. Materials and Methods

### 2.1. Study Area

This study area is the RER, which is located in northeastern Italy and covers 22,445 km^2^ [21]. The region has 4,438,937 inhabitants with a population density of 197.63 inhabitants/km^2^ [22]. The territory is formed by the Apennine Mountains (5677 km^2^; 25%), hills (6202 km^2^; 27%), and an alluvial plain (10,573 km^2^; 48%).

### 2.2. Case Definition 

The definition of a case of TL was based on the WHO directives [2,23]: a patient with suggestive cutaneous and/or mucosal lesion/s, in which *Leishmania* parasites were detected by histology and/or by molecular methods. Different TL types were defined following definitions that were established by the European LeishMan consortium [2]; CL refers to the presence of skin lesions, ML refers to the presence of mucosal lesion(s) without skin involvement, MCL refers to the simultaneous presence of both mucosal and skin lesions. Minor/mild disease was defined when lesions (papules or nodules) were less than 4, and small (<4 cm diameter), while the severe disease was identified when plaque/s was/were identified and/or 4 or more lesions were present and/or lesion diameter was 4 cm or above [23]. ML was always considered a severe disease. Lesion healing was defined as complete re-epithelialization for an ulcer or disappearance of induration for a papular lesion [2]. Treatment failure events were defined as relapse or persistence of lesions after 6-month treatment. Patients were usually followed up for 1 year after treatment.

TL cases were classified as: (i) autochthonous, when the place of infection was within RER; (ii) Italian extra-RER, when the place of infection was in Italy but outside RER; and (iii) imported, when the place of infection was outside Italy.

### 2.3. Surveillance of Tegumentary Leishmaniasis in Italy and in RER

Human leishmaniasis is a notifiable disease in Italy through an infectious disease reporting system, which is centralised at the national Ministry of Health [7,8]. Physicians are required to report all confirmed cases of leishmaniasis to the public health service of the Local Health Unit (LHU). The LHU carries out epidemiological investigations and collects data related to the infectious event; these data are subsequently sent to the regional health authorities and to the Ministry of Health [24]. In accordance with the RER surveillance program for leishmaniasis [19,20], molecular confirmation of TL is performed by the RRL, which is located at the Microbiology Unit of the University Hospital of Bologna. RRL receives biopsy samples from dermatologists, infectious disease specialists, and/or pathologists all over RER. 

### 2.4. Skin_Leish_RER Network

The Skin_Leish_RER network was established to improve the surveillance of TL cases in RER. Epidemiological data, clinical features, and data on anti-leishmanial therapy and outcome were collected. The network included ten diagnostic units: Piacenza, Parma, Reggio-Emilia, Modena, Bologna, Ferrara, Ravenna, Forlì, Cesena, and Rimini; these units include specialists in Parasitology, Dermatology, Infectious Diseases, and Pathology (Table S1). The Skin_Leish_RER network is led by the diagnostic unit at the University Hospital of Bologna, which comprises the RRL and the Dermatology Unit. 

### 2.5. Diagnosis of Tegumentary Leishmaniasis

Histological and molecular diagnosis were performed on skin or mucosal biopsies. For histological examination, biopsies were routinely formalin-fixed and paraffin-embedded (FFPE). Five- to six-µm sections were obtained from the block and stained with haematoxylin and eosin (HE) to detect leishmanial amastigotes. Slides were observed at 100×, 400×, and 600× magnification. If necessary, a Giemsa stain was evaluated in addition to HE. Molecular confirmation was performed on FFPE at RRL. DNA extraction was performed by the NucleoSpin DNA FFPE XS kit (Macherey-Nagel, Duren, Germany). Real-time PCR was carried out with two in-house assays, one targeting a segment of the small-subunit ribosomal RNA (rRNA) gene [25] and the other targeting a region of minicircle kinetoplast (k)DNA [26]. A real-time PCR assay targeting human β2-microglobulin was run simultaneously as a control of the amplification of the extracted DNA. PCR reactions were performed using a CFX real-time PCR detection system (Bio-Rad, Hercules, CA, USA) or Rotor-Q system (Qiagen, Hilden, Germany). Primers and probes employed for the real-time PCR assays are reported in Table S2. 

### 2.6. Data Collection

We collect all confirmed cases of TL (CL, ML) that were diagnosed in RER, for which biopsy samples were obtained between 1 January 2017 and 31 December 2020. Data were collected from the national notification system (SMI) and from the recovery activities of the Skin_Leish_RER network and organized in an electronic database that was shared with network members by using a password-protected platform. The sensible data of patients involved in this study were anonymized by an alphanumerical code. This study was conducted in accordance with the Declaration of Helsinki, and the protocol was approved by the Ethics Committee of the Area Vasta Emilia Centro (study number: EM1008-2021_97/2017/O/Tess/AOUBo). 

### 2.7. Statistical Analysis

We performed a descriptive analysis of TL cases by year of diagnosis, notification to LHU, origin of the case (autochthonous or imported), type of leishmaniasis, sex, age, molecular confirmation performed by RRL, clinical form, anatomical area of lesion/s, number of lesions, and presence of immunosuppressive conditions. The yearly incidence was evaluated for autochthonous cases. The seasonality of symptoms’ onset and delay in diagnosis were also assessed. All analyses were performed with Ms Excel 2017 and R 4.0.2 [27]. The spatial distribution of the autochthonous cases was obtained with QGis (version 3.14.15-Pi).

## 3. Results

### 3.1. Case Surveillance

Between January 2017 and December 2020, 135 cases of TL were identified in the RER, of which 113 (84%) were notified to the LHU (Table 1). The establishment of the Skin_Leish_RER network contributed to the recovery of 22 (16%) TL cases that were not reported to the public health authorities (Table 2). 

**Figure 1:**
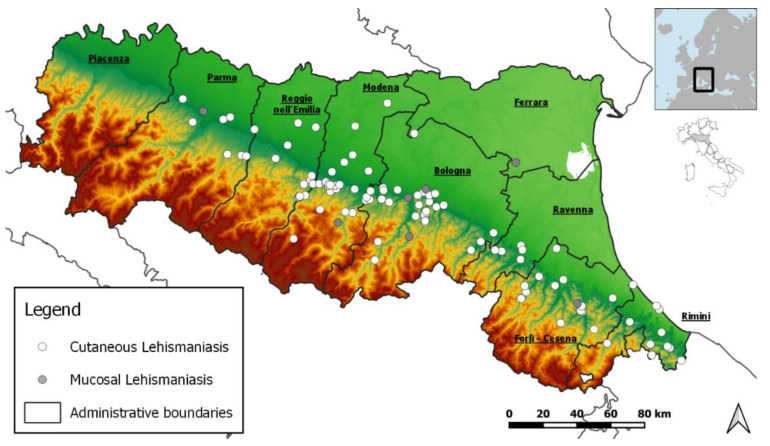
Geographic distribution of autochthonous cases of tegumentary leishmaniasis (n = 113), Emilia-Romagna region, northeastern Italy, 2017–2020.

Concerning the place of infection, 113 TL cases (84%) were classified as autochthonous (Table 1). Among the 22 non-autochthonous cases, the infection likely occurred in Italy for 14 cases, but outside the RER, the place of infection was likely outside Italy (Tunisia, Burkina Faso, and Morocco) in 5 cases. In the remaining cases (n = 3), the place of infection could not be defined. Autochthonous cases of TL were distributed in the foothills area of RER (Figure 1). 

**Figure 2:**
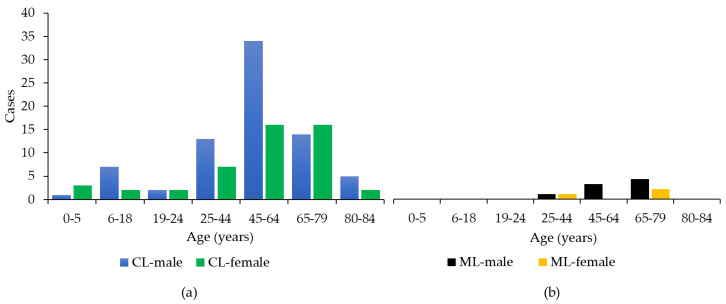
Age and sex distribution of cases of cutaneous (CL, **a**) and mucosal (ML, **b**) leishmaniasis (n = 135), Emilia-Romagna region (northeastern Italy), 2017–2020.

**Figure 3:**
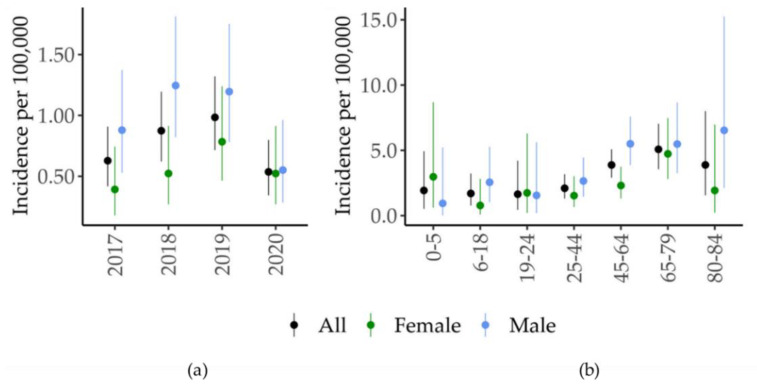
Incidence of tegumentary leishmaniasis by sex and year (**a**) and by age (**b**), Emilia-Romagna region (northeastern Italy), 2017–2020. CL, cutaneous leishmaniasis; ML, mucosal leishmaniasis.

Among the clinical forms of TL, the most represented was CL (n = 124, 92%), while ML was present in 11 cases (8%) (Table 1). Males were more affected than females (n = 84, 62%). The age of the affected patients ranged from 1 to 84 years, with a median age of 57 years; 75% of the cases were older than 43 years (Figure 2). In the study period, the incidence of TL rose from 0.63 in 2017 to 0.98 in 2019 and then dropped to 0.54 in 2020 (Figure 3a). The highest TL incidence was concentrated between 65 and 79 years (Figure 3b). No cases of MCL were detected in our study group. A clear seasonality in lesion onset was not evident (Figure S1, Kruskal-Wallis test *p* = 0.39). 

### 3.2. Clinicopathological Features

**Figure 4:**
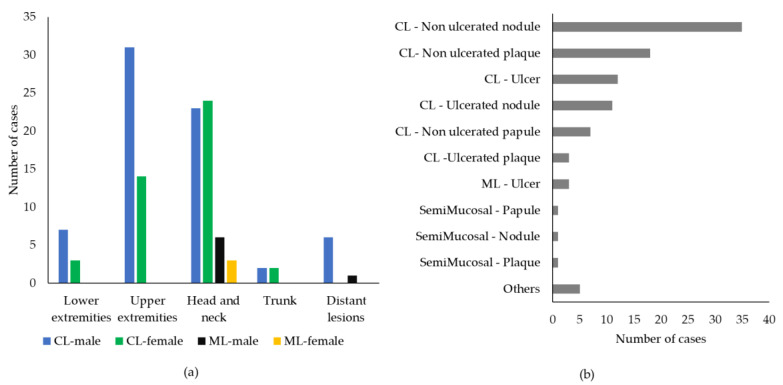
Clinical characteristics of tegumentary leishmaniasis cases in the Emilia-Romagna region, northeastern Italy, 2017–2020. (**a**) Frequency of lesions in different anatomic sites in males and females. Data were available for 124 cases of cutaneous leishmaniasis (CL) and 10 cases of mucosal leishmaniasis (ML); (**b**) frequency of different types of lesions. Data were available for 90 CL cases and 8 ML cases.

CL lesions were more often single (n = 88) than multiple (n = 19); for 17 CL cases, we had no information about the number of lesions. Concerning ML, five out of eleven cases (46%) presented multiple lesions, and five cases (46%) exhibited a single lesion, while no information was available for one case. As shown in Figure 4a, the most common location of cutaneous lesions was the head and neck (47 cases out of 124, 38%), followed by the upper (n = 45, 36%) and lower (n = 10, 8%) extremities, and trunk (n = 4, 3%). Six out of one hundred and twenty-four CL cases exhibited multiple lesions involving different and distant body sites (5%). ML mainly affected the head and neck (n = 9 out of 11, 82%), one HIV-positive patient exhibited multiple and distant lesions, and one case was not defined. Among ML cases, a previous episode of CL was reported by two patients (18%).

As shown in Figure 4b, data about the type of lesion were obtained for 98 out of 135 cases (n = 90 CL and n = 8 ML); the most common lesion type in CL was non-ulcerated nodules (n = 35 cases, 39%), followed by non-ulcerated plaques (n = 18, 20%), ulcers (n = 12, 13%), and ulcerated nodules (n = 11, 12%), while other lesion types, such as ulcerated plaques and non-ulcerated papules, were rare (Figure 4b). Regarding ML, lesions occurred as mucosal ulcers (n = 4, 50%), semi-mucosal nodule (n = 2, 25%), and plaques (n = 2, 25%), while data were not available for three cases. 

**Figure 5:**
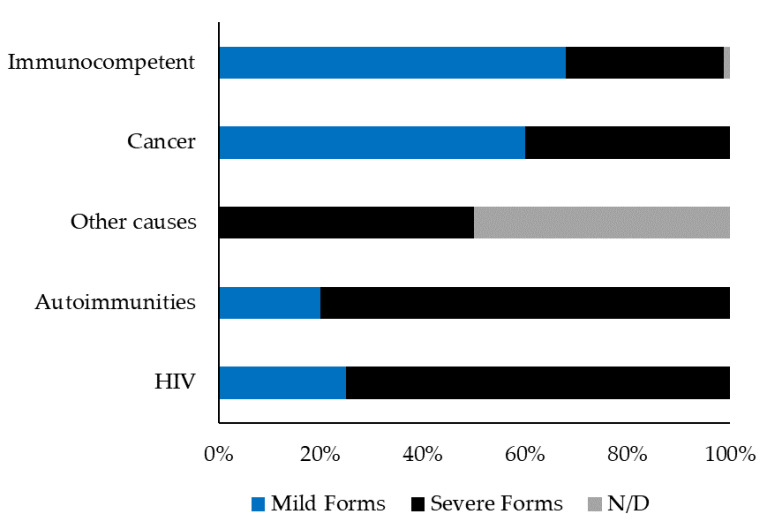
Comparative features of tegumentary leishmaniasis by patients’ immune status (n = 102). Severe and mild TL were defined in the Material and Method section. ND; not defined.

Information about potential immunosuppressive conditions was recorded for 102 patients; 16 individuals showed underlying conditions that could cause immune impairment, including HIV (4 patients), autoimmune diseases (5 cases), cancer (5 patients), and diabetes (1 case), while in one patient the immunosuppressive condition was undefined. As shown in Figure 5, among the 16 immunocompromised patients, 10 (63%) exhibited complicated TL, i.e., ML, presence of plaque, and/or multiple CL lesions, while only 26 out of 86 (30%) individuals with no impairment of the immune system exhibited severe TL. 

### 3.3. Diagnosis of Tegumentary Leishmaniasis 

Data on time to diagnosis were available for 92 patients; 36 cases of TL (39%) were diagnosed more than 6 months after the lesions’ onset (Table S3). 

**Figure 6:**
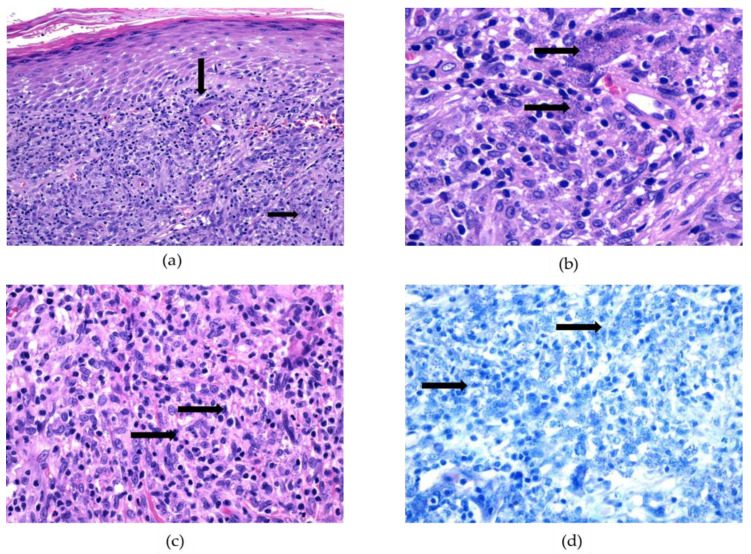
Histological diagnosis of tegumentary leishmaniasis: haematoxylin-eosin and Giemsa staining. (**a**,**b**): Hematoxylin-eosin (HE) staining reveals many amastigotes that appear as blue-greyish round structures, mostly contained in macrophages (black arrows), and that are associated with a dense inflammatory infiltrate and granulomatous areas. An abundance of amastigotes can be easily appreciated at low power view. (**a**): 100× magnification, (**b**): 600× magnification. (**c**,**d**): When the amastigotes are scarce, or the inflammatory infiltrate is particularly intense, the diagnosis of leishmaniasis can be overlooked by employing HE staining (**c**, 400× magnification); in these cases, Giemsa staining (**d**) can contribute to revealing the amastigotes (black arrows, 400× magnification).

The diagnosis of TL was carried out by histology and/or molecular tools. Histological examination was carried out in 118 out of 135 cases (Figure 6 and Table S4); among these, intracytoplasmic amastigotes were detected in 92 cases with a sensitivity of 78%. PCR was performed in 103 out of 135 cases; 100 cases tested positive for leishmanial DNA, showing a sensitivity of 97%. We observed that 11 of the 22 non-notified cases (50%) were not sent for molecular confirmation to RRL. 

### 3.4. TL Treatment and Outcome

**Figure 7:**
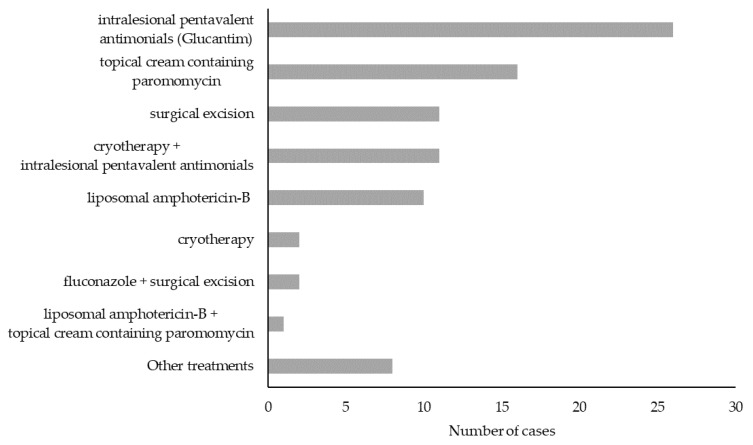
Therapeutic approaches for tegumentary leishmaniasis in the Emilia-Romagna region, northeastern Italy, 2017–2020.

Data on treatment were recorded in 90 out of 135 (66%) cases (Figure 7). Therapy options included (i) intralesional pentavalent antimonial (Glucantim) (n = 27, 30%), (ii) topical cream containing paromomycin (n = 16, 18%), (iii) surgical excision (n = 13, 14%), (iv) cryotherapy alone (n = 2, 2%) or associated with intralesional Glucantim (n = 11, 12%), (v) systemic liposomal amphotericin-B alone (n = 11, 12%), (vi) other treatments (n = 10, 11%) such as oral fluconazole (n = 2), oral miltefosine (n = 1), surgical excision associated with fluconazole (n = 1), intralesional Glucantim associated with miltefosine (n = 2), intralesional Glucantim associated with liposomal amphotericin-B (n = 2), or intralesional Glucantim associated with cryotherapy, and intramuscular pentamidine (n = 2).

Data on treatment outcome were available for 84 out of 135 cases (62%, Figure S2). Among these, treatment failure events were observed in 18 cases (14 CL and 4 ML, 21%). By considering only TL cases for which treatment outcome was available, the failure rate with different therapeutic approaches was as follows: 7 cases out of 11 for liposomal amphotericin-B alone (64%), 4 cases out of 11 for surgical excisions (36%), 3 cases out of 14 for topical cream containing paromomycin (21%), and 1 case out of 26 for intralesional Glucantim (4%). Among the cases of treatment failure, 5 (28%) occurred in immunocompromised patients (2 HIV-positive individuals and 3 oncological patients), 6 (33%) in immunocompetent individuals, while in 7 patients (39%) the immune status was not defined.

## 4. Discussion 

Leishmaniases are considered endemic and emerging infections in southern and continental Europe, respectively, yet these infections are underrecognized and underreported in most EU countries [7]. Despite being more frequent than VL, TL cases are often overlooked; the lack of awareness about this infectious disease by dermatologists, otorhinolaryngologists, and other clinicians often results in misdiagnosis. Even when a proper diagnosis of TL is performed, case notification to the public health services is frequently lacking. 

Our study examined TL cases that were identified in an endemic area of 4.4 million inhabitants in northeastern Italy. The retrospective collection of cases between 2017 and 2020 was implemented by the establishment of a multidisciplinary network of medical specialists (Skin_Leish_RER network). The network recovered 22 cases beyond the 113 notified cases, for a total of 135 confirmed cases of TL in a 4-year period, with a yearly incidence ranging from 0.54 to 0.98 cases per 100,000 inhabitants. The drop in TL cases in 2020 (0.54 cases/100,000 inhabitants) was likely due to the disruption of the health system due to the COVID pandemic [28].

Most of the TL cases in the selected region were autochthonous (84% of total cases) and distributed in foothills areas, which are characterized by climatic and environmental conditions more suitable for the occurrence of phlebotomine sand flies as compared to the plain areas of the RER [29]. Indeed, foothill habitats have previously been reported as supporting large populations of *Phlebotomus (Ph). perfiliewi*, a sand fly species able to maintain the circulation of *L. infantum* [30,31,32]. The geographic area of TL distribution overlaps with that of VL, which also showed an increasing number of cases in the last decade [14,17,18,33].

In line with published evidence [2], most patients with TL caused by *L. infantum* were male and older than 43 years; the predominant clinical feature was a single cutaneous nodule in the head, neck or in the upper extremities. Further, we observed that 8% of TL cases were ML; in line with recent findings [2] *L. infantum* can disseminate to the mucous membranes with a higher frequency than previously reported [11]. Besides the immune condition of the host and vectorial factors, the tissue tropism of *L. infantum* could be linked to the intrinsic virulence of the parasite [34] and/or strain genotype [35]. Molecular typing on TL specimens is ongoing and could provide additional information on this matter.

Among the 16 immunocompromised patients, 10 (63%) exhibited a severe disease, i.e., ML and/or more than 4 CL lesions and/or a lesion’s diameter > 4 cm and/or plaques, while only 26 (31%) out of 84 individuals with no underlying immunosuppressive conditions exhibited a severe TL. Despite data being available only for 84 individuals, the treatment failure rate was also higher in immunocompromised patients than in immunocompetent individuals (45% vs. 10%, respectively). These findings are not surprising, as immunocompromised patients are predisposed to develop multiple lesions with a higher frequency of ML and exhibit higher rates of treatment failure [36]. 

Case confirmation of TL relies on the direct demonstration of the parasite or its genome in clinical specimens from the skin or mucous membranes, using microscopic examination or molecular analysis, respectively [23]. It is widely recognized that molecular methods are highly sensitive and specific tools to detect leishmanial DNA in dermal biopsies [37,38,39,40]. Considering the 86 samples examined with PCR and histology in this retrospective study, we observed that real-time PCR allowed the detection of 26 (30%) cases of TL that histology failed to identify. Even though this study was not designed to compare histology and molecular methods for the diagnosis of TL, PCR seems to have better performance than the direct detection of parasites by histopathology in biopsies. 

Differently from other European countries, including France [41], there are no national guidelines for the treatment of TL in Italy. We counted twelve different treatment strategies that were employed by the physicians belonging to the Skin_Leish_RER network, including pharmacological therapies, cryotherapy, or surgical excision. Local treatment was employed in most cases (56 out of 90, 62%), while systemic treatment alone or associated with local treatment was used in 23% of cases. These data are in line with international reports and guidelines indicating that local pharmacological and/or physical treatment can be employed for non-complicated CL in southern Europe [2,23,41]. In the remaining cases (n = 13, 14%) surgical excision was carried out, and it was associated with a 36% relapse rate. Surgery is not recommended for CL treatment but is sometimes performed with the erroneous suspicion of skin cancer. Taken together, the abovementioned observations suggest that standardization of TL treatment is needed, including the development of national guidelines. 

This study has several limitations. By collecting data from a limited geographic area, the results cannot be generalized to the entire country. Further, because of the retrospective nature of this study, information regarding significant variables, including follow-up data, were often lacking. In addition, HIV testing was not systematically performed, and the condition of immunosuppression was not always recorded; therefore, the actual prevalence of TL in immunocompromised patients in northeastern Italy cannot be defined. 

## 5. Conclusions

We provided evidence of a high incidence of TL with a specific geographic pattern in RER, a region of northeastern Italy, as well as a relevant number of ML cases. Our observations suggest the need to raise awareness about TL among clinicians and pathologists, promote the molecular confirmation of cases by reference laboratories, and encourage the establishment of surveillance networks for this neglected disease.

## Figures and Tables

**Table 1:** Tegumentary leishmaniasis cases that were identified in the Emilia-Romagna region, northeastern Italy, 2017–2020.

Year	TL Cases (n.)	Notified to LHU n. (%)	Autochthonous Cases n. (%)	CL Cases n. (%)	ML Cases n. (%)
2017	28	21 (75%)	21 (75%)	23 (82%)	5 (18%)
2018	39	36 (92%)	34 (87%)	38 (97%)	1 (3%)
2019	44	38 (86%)	40 (91%)	41 (93%)	3 (7%)
2020	24	18 (75%)	18 (75%)	22 (92%)	2 (8%)
Total	135	113 (84%)	113 (84%)	124 (92%)	11 * (8%)

* 7 autochthonous cases and 4 cases in which the place of infection was defined outside RER, within Italy.

**Table 2:** Tegumentary leishmaniasis: case notification and/or molecular confirmation at the Regional Reference Laboratory (RRL), Emilia-Romagna region, Italy.

	Case Notification		
		Yes	No
**PCR Confirmation**	**Yes**	92 (68%)	11 (8%)
	**No**	21 (15%)	11 (8%)
Total		113 (84%)	22 (16%)

In brackets: percentages are calculated based on total cases (n = 135) of tegumentary leishmaniasis.

## Data Availability

Data are available from the corresponding author (SV) upon reasonable request.

## Supplementary Materials

The following supporting information can be downloaded at: https://www.mdpi.com/article/10.3390/ijerph192316047/s1, Table S1. Network of diagnostic centres for the surveillance of tegumentary leishmaniasis, Emilia-Romagna region (northeastern Italy), 2017–2020. Table S2. Primers and probes employed for molecular diagnosis of tegumentary leishmaniasis (real-time PCR). Table S3. Delay in the diagnosis and notification status of tegumentary leishmaniasis cases in the Emilia-Romagna region (northeastern Italy) (n = 92). Table S4. Diagnosis of tegumentary leishmaniasis by histology (n = 118) and/or by PCR (n = 103). Figure S1. Seasonal distribution of tegumentary leishmaniasis based on lesions’ onset (n = 85 TL cases). Figure S2. Treatment failure in different therapeutic approaches for n=84 cases of tegumentary leishmaniasis in the Emilia-Romagna region, northeastern Italy, 2017–2020.

## References

[1] Gradoni L. (2013). Epidemiological surveillance of leishmaniasis in the European Union: Operational and research challenges. Euro Surveill.

[2] Guery R., Walker S.L., Harms G., Neumayr A., Van Thiel P., Gangneux J.P., Clerinx J., Söbirk S.K., Visser L., Lachaud L. (2021). Clinical diversity and treatment results in Tegumentary Leishmaniasis: A European clinical report in 459 patients. PLoS Negl. Trop. Dis..

[3] Grifferty G., Shirley H., McGloin J., Kahn J., Orriols A., Wamai R. (2021). Vulnerabilities to and the Socioeconomic and Psychosocial Impacts of the Leishmaniases: A Review. Res. Rep. Trop Med..

[4] Gianchecchi E., Montomoli E. (2020). The enemy at home: Leishmaniasis in the Mediterranean basin, Italy on the focus. Expert Rev. Anti-Infect. Ther. Aims Scope.

[5] Ruiz-Postigo J.A., Grout L., Jain S. (2020). Global Leishmaniasis Surveillance, 2017–2018, and First Report on 5 Additional Indicators.

[6] Ruiz-Postigo R.-P., Saurabh J., Alexei M., Maia-Elkhoury M.-E.A.N., Samantha V., Supriya W., Mona O., Zaw L., Abate B., Aya Y. (2021). Global Leishmaniasis Surveillance: 2019–2020, A Baseline for the 2030 Roadmap.

[7] Berriatua E., Maia C., Conceição C., Özbel Y., Töz S., Baneth G., Pérez-Cutillas P., Ortuño M., Muñoz C., Jumakanova Z. (2021). Leishmaniases in the European Union and Neighboring Countries. Emerg. Infect. Dis..

[8] Alvar J., Vélez I.D., Bern C., Herrero M., Desjeux P., Cano J., Jannin J., den Boer M., Team W.L.C. (2012). Leishmaniasis worldwide and global estimates of its incidence. PLoS ONE.

[9] Gaspari V., Zaghi I., Macrì G., Patrizi A., Salfi N., Locatelli F., Carra E., Re M.C., Varani S. (2020). Autochthonous Cases of Mucosal Leishmaniasis in Northeastern Italy: Clinical Management and Novel Treatment Approaches. Microorganisms.

[10] Santi A., Renzi M., Baldelli R., Calzolari M., Caminiti A., Dell’Anna S., Galletti G., Lombardini A., Paternoster G., Tamba M. (2014). A surveillance program on canine leishmaniasis in the public kennels of Emilia-Romagna Region, Northern Italy. Vector-Borne Zoonotic Dis..

[11] Faucher B., Pomares C., Fourcade S., Benyamine A., Marty P., Pratlong L., Faraut F., Mary C., Piarroux R., Dedet J.P. (2011). Mucosal *Leishmania infantum* leishmaniasis: Specific pattern in a multicentre survey and historical cases. J. Infect..

[12] WHO Italy. http://who-dev.essi.upc.edu/who/leishmaniasis.html.

[13] Maroli M., Rossi L., Baldelli R., Capelli G., Ferroglio E., Genchi C., Gramiccia M., Mortarino M., Pietrobelli M., Gradoni L. (2008). The northward spread of leishmaniasis in Italy: Evidence from retrospective and ongoing studies on the canine reservoir and phlebotomine vectors. Trop. Med. Int. Health.

[14] Mattivi A., Massimiliani E., Cagarelli R., Albieri A. LEISHMANIOSI IN EMILIA-ROMAGNA, Aggiornamento Epidemiologico 1999–2015. https://salute.regione.emilia-romagna.it/normativa-e-documentazione/rapporti/malattie-infettive/leishmaniosi-er-epidemiologia-1999-2015.

[15] Gaspari V., Ortalli M., Foschini M.P., Baldovini C., Lanzoni A., Cagarelli R., Gaibani P., Rossini G., Vocale C., Tigani R. (2017). New evidence of cutaneous leishmaniasis in north-eastern Italy. J. Eur. Acad Dermatol. Venereol..

[16] Cesinaro A.M., Nosseir S., Mataca E., Mengoli M.C., Cavatorta C., Gennari W. (2017). An outbreak of cutaneous leishmaniasis in Modena province (Northern Italy): Report of 35 cases. Pathologica.

[17] Varani S., Cagarelli R., Melchionda F., Attard L., Salvadori C., Finarelli A.C., Gentilomi G.A., Tigani R., Rangoni R., Todeschini R. (2013). Ongoing outbreak of visceral leishmaniasis in Bologna Province, Italy, November 2012 to May 2013. Euro Surveill.

[18] Franceschini E., Puzzolante C., Menozzi M., Rossi L., Bedini A., Orlando G., Gennari W., Meacci M., Rugna G., Carra E. (2016). Clinical and Microbiological Characteristics of Visceral Leishmaniasis Outbreak in a Northern Italian Nonendemic Area: A Retrospective Observational Study. Biomed Res. Int..

[19] Regione Emilia-Romagna Individuazione Del Laboratorio di Riferimento Regionale Per la Diagnosi di Leishmaniosi Viscerale e Cutanea Umana. https://salute.regione.emilia-romagna.it/sanita-pubblica/malattie-infettive/nota-regionale-luglio-2014.pdf/@@download/file/nota%20regionale%20luglio%202014.pdf.

[20] Regione Emilia-Romagna Individuazione del Laboratorio di Riferimento Regionale Per la Diagnosi di Leishmaniosi Viscerale e Cutanea Umana. Precisazioni e Integrazioni Alla Nota PG_2014_276720. https://salute.regione.emilia-romagna.it/sanita-pubblica/malattie-infettive/pg_2014_373414-completo_.pdf/@@download/file/PG_2014_373414%20completo_.pdf.

[21] ISTAT Superfici Territoriali. http://dati.istat.it/Index.aspx?DataSetCode=DCCV_CARGEOMOR_ST_COM.

[22] ISTAT Popolazione Residente al 1° Gennaio 2021: Emilia-Romagna. http://dati.istat.it/index.aspx?queryid=18560.

[23] Gradoni L., López-Vélez R., Mokni M. (2017). Manual on Case Management and Surveillance of the Leishmaniases in the WHO European Region.

[24] Italian Ministry of Health Prevenzione e Controllo Della Leishmaniosi in Italia. https://www.trovanorme.salute.gov.it/norme/renderNormsanPdf?anno=2020&codLeg=77839&parte=1%20&serie=null.

[25] Wortmann G., Sweeney C., Houng H.S., Aronson N., Stiteler J., Jackson J., Ockenhouse C. (2001). Rapid diagnosis of leishmaniasis by fluorogenic polymerase chain reaction. Am. J. Trop. Med. Hyg..

[26] Mary C., Faraut F., Lascombe L., Dumon H. (2004). Quantification of *Leishmania infantum DNA* by a real-time PCR assay with high sensitivity. J. Clin. Microbiol..

[27] R Core Team (2022). A Language and Environment for Statistical Computing.

[28] Spadea T., Di Girolamo C., Landriscina T., Leoni O., Forni S., Colais P., Fanizza C., Allotta A., Onorati R., Gnavi R. (2021). Indirect impact of Covid-19 on hospital care pathways in Italy. Sci. Rep..

[29] Calzolari M., Romeo G., Munari M., Bonilauri P., Taddei R., Sampieri M., Bariselli S., Rugna G., Dottori M. (2022). Sand Flies and Pathogens in the Lowlands of Emilia-Romagna (Northern Italy). Viruses.

[30] Calzolari M., Angelini P., Finarelli A.C., Cagarelli R., Bellini R., Albieri A., Bonilauri P., Cavrini F., Tamba M., Dottori M. (2014). Human and entomological surveillance of Toscana virus in the Emilia-Romagna region, Italy, 2010 to 2012. Euro Surveill.

[31] Calzolari M., Carra E., Rugna G., Bonilauri P., Bergamini F., Bellini R., Varani S., Dottori M. (2019). Isolation and Molecular Typing of of *Leishmania infantum* from *Phlebotomus perfiliewi* in a Re-Emerging Focus of Leishmaniasis, Northeastern Italy. Microorganisms.

[32] Calzolari M., Romeo G., Callegari E., Bonilauri P., Chiapponi C., Carra E., Rugna G., Taddei R., Lelli D., Dottori M. (2021). Co-Circulation of Phleboviruses and Leishmania Parasites in Sand Flies from a Single Site in Italy Monitored between 2017 and 2020. Viruses.

[33] Rugna G., Carra E., Bergamini F., Calzolari M., Salvatore D., Corpus F., Gennari W., Baldelli R., Fabbi M., Natalini S. (2018). Multilocus microsatellite typing (MLMT) reveals host-related population structure in *Leishmania infantum* from northeastern Italy. PLoS Negl. Trop. Dis..

[34] Ait Maatallah I., Akarid K., Lemrani M. (2022). Tissue tropism: Is it an intrinsic characteristic of Leishmania species?. Acta Trop..

[35] Chargui N., Amro A., Haouas N., Schönian G., Babba H., Schmidt S., Ravel C., Lefebvre M., Bastien P., Chaker E. (2009). Population structure of Tunisian *Leishmania infantum* and evidence for the existence of hybrids and gene flow between genetically different populations. Int. J. Parasitol..

[36] van Griensven J., Carrillo E., López-Vélez R., Lynen L., Moreno J. (2014). Leishmaniasis in immunosuppressed individuals. Clin. Microbiol. Infect..

[37] de Vries H.J., Reedijk S.H., Schallig H.D. (2015). Cutaneous leishmaniasis: Recent developments in diagnosis and management. Am. J. Clin. Dermatol..

[38] Tordini G., Giaccherini R., Pacenti L., Miracco C., Zazzi M., Zanelli G. (2007). Cutaneous leishmaniasis: Usefulness of PCR on paraffin-embedded skin biopsies as part of routine investigation. Ann. Trop. Med. Parasitol..

[39] Martín-Ezquerra G., Fisa R., Riera C., Rocamora V., Fernández-Casado A., Barranco C., Serra T., Baró T., Pujol R.M. (2009). Role of *Leishmania* spp. infestation in nondiagnostic cutaneous granulomatous lesions: Report of a series of patients from a Western Mediterranean area. Br. J. Dermatol..

[40] Silgado A., Armas M., Sánchez-Montalvá A., Goterris L., Ubals M., Temprana-Salvador J., Aparicio G., Chicharro C., Serre-Delcor N., Ferrer B. (2021). Changes in the microbiological diagnosis and epidemiology of cutaneous leishmaniasis in real-time PCR era: A six-year experience in a referral center in Barcelona. PLoS Negl. Trop. Dis..

[41] Morizot G., Kendjo E., Mouri O., Thellier M., Pérignon A., Foulet F., Cordoliani F., Bourrat E., Laffitte E., Alcaraz I. (2013). Travelers with cutaneous leishmaniasis cured without systemic therapy. Clin. Infect. Dis..

