# Supplementary material for: Tegumentary Leishmaniasis in Northeastern Italy from 2017 to 2020: A Neglected Public Health Issue
Source: Int J Environ Res Public Health. 2022 Nov 30;19(23):16047. doi: 10.3390/ijerph192316047 (PMC9740434; doi:10.3390/ijerph192316047)
Supplement: Supplementary file 1 [file ijerph-19-16047-s001.zip › ijerph-2034864-supplementary.pdf]

Supplementary Material

Table S1. Network of diagnostic centres for the surveillance of tegumentary leishmaniasis, Emilia-Romagna region (northeastern Italy), 2017-2020.

Diagnostic Unit	Hospital Name	Number of cases
Piacenza	Azienda USL di Piacenza-Ospedale Civile Piacenza	1
Parma	Azienda Ospedaliero-Universitaria di Parma	13
Reggio Emilia	Azienda USL- IRCCS Reggio Emilia-Arcispedale Santa Maria Nuova	10
Modena	Azienda Ospedaliero-Universitaria Policlinico di Modena	40
Bologna	IRCCS/Azienda Ospedaliero-Universitaria di Bologna	35
Ferrara	Azienda Ospedaliero-Universitaria di Ferrara	0
Ravenna	Azienda USL della Romagna-Ospedale di Ravenna	5
Forlì	Azienda USL della Romagna-Ospedale Morgagni Forlì	6
Cesena	Azienda USL della Romagna-Ospedale Bufalini di Cesena	12
Rimini	Azienda USL della Romagna-Ospedale di Rimini	13
Total number of cases, 2017-2020		135

Table S2. Primers and probes employed for molecular diagnosis of tegumentary leishmaniasis (real-time PCR).

Gene target	Primer sequences (Forward-Reverse)	Probe sequences
Small-subunit ribosomal RNA (rRNA) gene	5′-AAGTGCTTTCCCATCGCAACT-3′<br>5′-GACGCACTAAACCCCTCCAA-3′	5′-FAM-CGGTTCGGTGTGTGGCGCC-3′
Kinetoplast (k)DNA	5′-CTTTTCTGGTCCTCCGGGTAGG-3′<br>5′-CCACCCGGCCCTATTTTACACCAA-3′	5′-FAM-TTTTCGCAGAACGCCCCTACCCGC-3′
β2-microglobulin (housekeeping gene)	5′-TGAGTATGCCTGCCGTGTGA-3′<br>5′-ACTCATACACAACTTTCAGCAGCTTAC-3′	5′- FAM-CCATGTGACTTTGTCACAGCCCAAGATAGTT-3′

Table S3. Delay in the diagnosis and notification status of tegumentary leishmaniasis cases in the Emilia-Romagna region (northeastern Italy) (n=92).

**Lesion onset/diagnosis time gap**	**Public health notification**		**Total cases (%)**
	**yes**	**no**	
**<= 1 month**	9	0	9 (10%)
**1-3 months**	11	4	15 (16%)
**3-6 months**	26	6	32 (35%)
**6-12 months**	28	2	30 (33%)
**>12 months**	6	0	6 (7%)
**Total**	80	12	92 (100%)

Table S4. Diagnosis of tegumentary leishmaniasis by histology (n=118) and/or by PCR (n=103).

		Histology			
		**Amastigotes +**	**Amastigotes -**	**Not done**	**Total**
**Molecular diagnosis**	PCR+	57	26	17*	100
	PCR-	3	0	0	3
	Not done	32	0	0	32
	Total	92	26	17	

*11 out of 17 cases were not reported to the public health service.

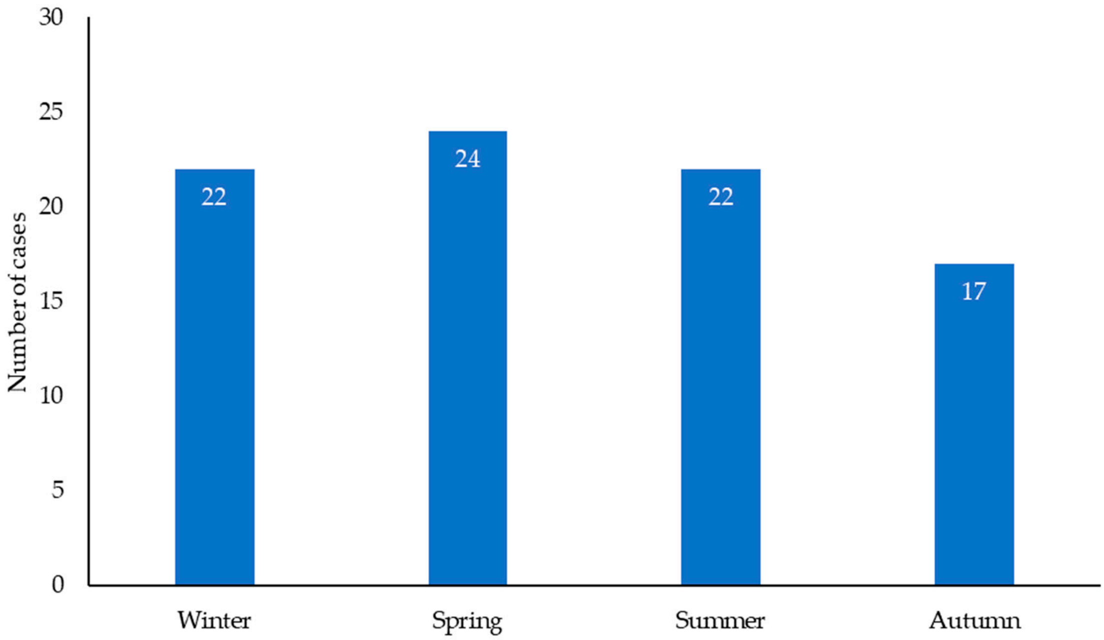

Figure S1. Seasonal distribution of tegumentary leishmaniasis based on lesions onset. Data on lesion onset were available for n= 85 TL cases.

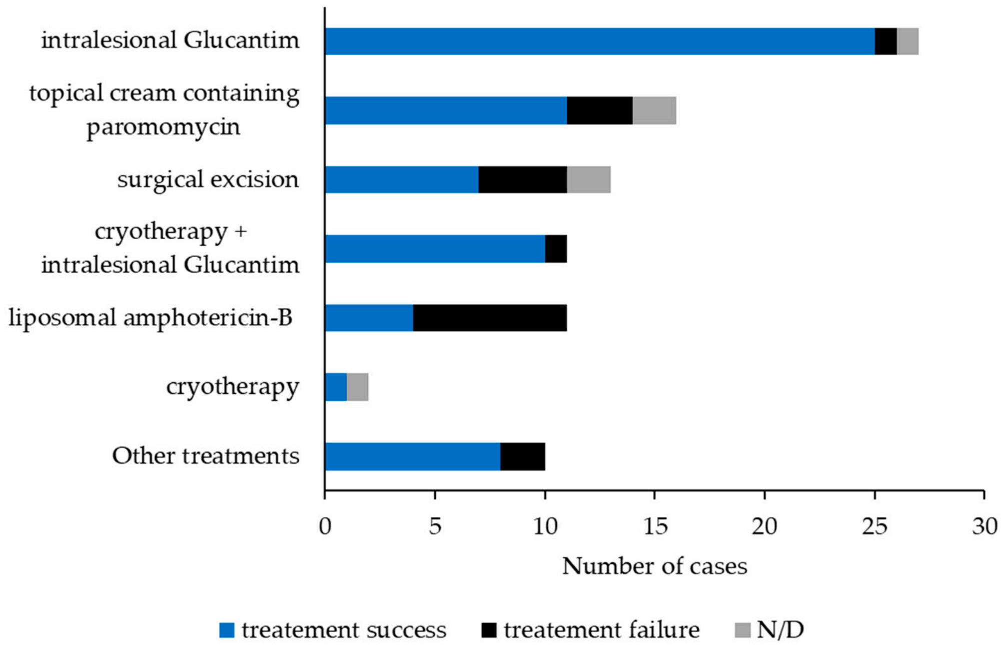

Figure S2. Treatment failure in different therapeutic approaches for n=84 cases of tegumentary leishmaniasis in the Emilia-Romagna region, northeastern Italy, 2017-2020. ND; not defined
